# Comparative efficacy and safety of injection therapies for knee osteoarthritis: A protocol for systematic review and Bayesian network meta analysis

**DOI:** 10.1097/MD.0000000000022943

**Published:** 2020-11-20

**Authors:** Ting Yu, ShiFan Yan, ZhenHai Chi, DaoCheng Zhu, Pan Cheng, HaiYan Li, SiYu Qin, GenPing Zhong, XiLin Ouyang, RiXin Chen, Lin Jiao

**Affiliations:** a Jiangxi University of Traditional Chinese Medicine; b Affiliated Hospital of Jiangxi University of Traditional Chinese Medicine, Nanchang, China.

**Keywords:** injection therapy, knee osteoarthritis, protocol, systematic review

## Abstract

**Introduction:**

There are many injection methods for the treatment of knee osteoarthritis, but there is no comprehensive comparison, based on the fixed effect model.

**Methods:**

According to the retrieval strategy, we searched randomized controlled trials (RCTs) randomly from PubMed, the Cochrane Library, Embase, the China National Knowledge Infrastructure (CNKI), Wanfang Database from their inceptions to August 2020, and 2 members of us selected literatures and extracted data independently. Methodological quality was assessed by using the Cochrane bias risk tool, and meta-analysis was performed by using the Stat.14.0.

**Results:**

This study will evaluate the effectiveness and safety of different injectable drugs for the treatment of knee osteoarthritis and rank the efficacies of drugs, then to determine the optimal treatment.

**Conclusion:**

This study will provide evidence for the choice of injection therapy for knee osteoarthritis.

**INPLASY registration number:**

INPLASY202080099.

## 1 Introduction

Knee osteoarthritis is a common chronic degenerative disease, which is mostly occurring in the elderly. Its main clinical symptoms include knee pain, impaired activity, late stage, and even disability, which seriously affects daily life and work.^[1,2]^ According to epidemiology, the global prevalence of KOA is 12% to 35%, and currently, there are about 250 million people who are suffering by knee osteoarthritis.^[3,4]^

Currently, the treatment methods for knee osteoarthritis can be divided into surgical treatment and conservative treatment. Due to economic problems, surgical risks, postoperative complications, and other reasons, most patients choose conservative treatment. Conservative treatment mainly includes oral or external anti-inflammatory drugs (nonsteroidal anti-inflammatory drugs), intra-articular (IA) injection, exercise therapy, physical therapy, etc.^[5,6]^ IA injection has been proved to be effective in relieving pain and improving function in many studies.^[7–9]^ However, injection therapy includes many drugs, such as hyaluronic acid, ozone, platelet-rich plasma (PRP), botulinum toxin (BT), and corticosteroids. The questions are, will different drugs have different effects? Which drug is the best choice for treating knee osteoarthritis?

In recent years, many scholars have carried out randomized controlled trials (RCTs) studies on these drugs, and many relevant systematic evaluations and meta-analyses had been published based on clinical research results, but most meta-analyses^[9–11]^ only compared one treatment approach with blank control or another. On the contrary, in the previous network meta-analysis, scholars^[12]^ only compared the therapeutic effects of 4 IA injection therapies on knee osteoarthritis, without mentioning ozone, BT, and other treatments.

Considering the above-mentioned issues and the recent publication of a number of new RCTs,^[13]^ we believe that it is necessary to conduct a meta-analysis of the Network to explore the efficacy of various treatment methods in the treatment of knee osteoarthritis, so that to comprehensively evaluate the safety and efficacy of injection therapy and provide evidence for the clinical treatment of knee osteoarthritis.

## 2 Methods

### 2.1 Inclusion criteria

#### 2.1.1 Type of studies

All interventions, including at least 2 RCT injections for knee osteoarthritis, should be selected, with injections limited to BT, corticosteroid (CS), hyaluronate (HYA), peppering technique (PEP), PRP, placebo (PLA).

#### 2.1.2 Type of participant

According to the diagnostic criteria of knee osteoarthritis stipulated in guidelines for the “Diagnosis and Treatment of Osteoarthritis,”^[16]^ “Practical Osteology,”^[17]^ “American College of Rheumatology (1987),”^[18]^ etc, the main contents are as follows: Recurrent knee pain in the past 1 month; Accompanied by any of the following at least 2 cases, X-rays in standing or weight-bearing positions showed narrowing of joint space, subchondral osteosclerosis or cystic changes, and joint marginal osteophyte formation; More than 50 years old; Joint stiffness time is less than 30 minutes after get up in the morning; Sound of bone friction or bone friction when the joints move.

#### 2.1.3 Types of intervention

The interventions under study should include at least 2 different injection therapeutics, limited to botulinum toxin (BT), CS, HYA, peppering technique (PEP), placebo and ’wait and see’ (PLA), and bright-rich plasma (PRP). They were excluded from ozone therapy, Autologous Blood (AB), glycosaminoglycan polysulfate (GSGPS), Prolotherapy (PRO), stem cell therapy, etc.

#### 2.1.4 Types of outcome measurements

##### 2.1.4.1 Primary outcome

Western Ontario and McMaster Universities Osteoarthritis Index (WOMAC) is used to evaluate the structure and function of the knee from the 3 aspects of symptoms and signs of pain, stiffness, and joint function, and evaluate the treatment effect through the score changes before and after treatment.

##### 2.1.4.2 Secondary outcomes

(1) Visual analog scale (VAS) for pain, A ruler marked with a number from 1 to 10 indicates pain, 0 is painless, and 10 is the ultimate tolerable pain. The patient marks his or her own pain on the ruler.
(2) Scoring for Lysholm knee makes a preliminary assessment from the levels of different levels of exercise function, the rating is more inclined to the daily life activities.
(3) Aims2-s is used to evaluate patients from 5 aspects, including body, symptoms, and emotions. There are total of 20 items, which are often used to evaluate patients’ quality of life.
(4) The incidence rate of adverse events.

#### 2.1.5 Exclusion criteria

(1) Exclusion of reviews, animal experiments, case reports, and non-RCTs;
(2) The comparison of efficacy between different doses or injection sites of only one injection therapy is excluded.
(3) If there is any information loss or obvious error in the article, it will be screened out for further study.

### 2.2 Search methods for identification of studies

Comprehensive searches of RCTS on injection therapy for knee osteoarthritis were conducted in 3 English databases of PubMed, Cochrane Library, Embase, and 2 Chinese databases of CNKI and Wanfangs, and the time of index was from their inceptions to August 2020 for each database. The retrieval strategy of PubMed is summarized in Table 1.

**Table 1:** Search strategy used in PubMed database.

Number	Search items
#1	randomized controlled trial [pt]
#2	controlled clinical trial [pt]
#3	randomized [tiab]
#4	clinical trials as topic [mesh: noexp]
#5	randomly [tiab]
#6	trial [ti]
#7	OR/ #1–#6
#8	Knee Osteoarthritides [Mesh]
#9	Knee Osteoarthritis [All Fields)
#10	Osteoarthritis of Knee [All Fields)
#11	Osteoarthritis of the Knee [All Fields)
#12	OR/#8–#11
#13	Hyaluronic acid [Mesh]
#14	Vitrax, Amo OR Biolon OR Etamucine OR Hyaluronan OR Hyvisc OR Luronit OR Sodium Hyaluronate OR Hyaluronate, Sodium OR Hyaluronate Sodium OR Amvisc OR Healon [All Fields)
#15	OR/#13–#14
#16	Platelet-Rich Plasma [Mesh]
#17	Plasma, Platelet-Rich [All Fields)
#18	Platelet Rich Plasma [All Fields)
#19	OR/#16–#18
#20	Botulinum Toxin [Mesh]
#21	Toxins, Botulinum [All Fields)
#22	Toxin, Botulinum [All Fields)
#23	Clostridium botulinum Toxins [All Fields)
#24	Toxins, Clostridium botulinum [All Fields)
#25	Botulin [All Fields)
#26	OR/#20–#25
#27	Adrenal Cortex Hormones [Mesh]
#28	Hormones, Adrenal Cortex [All Fields)
#29	Corticosteroids [All Fields)
#30	Corticoids [All Fields)
#31	OR/#27–#30
#32	#15 OR #19 OR #26 OR #31
#33	7 AND #12 AND #32

### 2.3 Data collection

#### 2.3.1 Selection of studies

**Figure 1:**
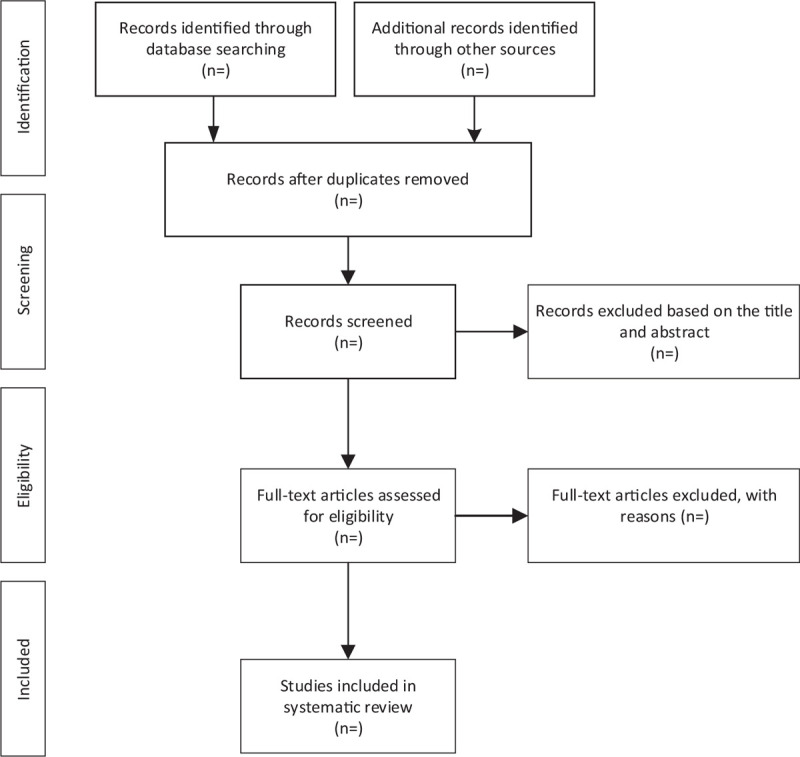
Flow diagram of study selection process.

For the convenience of management, we imported the titles retrieved from the 5 databases into EndNote Software AQ8 (V.X9); we deleted the duplicate articles, 2 team members (YT and YSF) of us independently read the titles and abstractions, and deleted the nonconforming literatures, then read the full text of articles that cannot be identified by the title and identify the ones that will eventually be included. At the end of this step, the results of both parties are cross-checked, if any objections arise, a decision is made through group discussion and will be decided by a third reviewer (JL). The overall process and results are shown in Figure 1.

#### 2.3.2 Data extraction and management

Microsoft Excel 2016 was used to establish information data extraction table, and pre-extraction was carried out to determine the feasibility of the table. Then, 2 team members (YT and YSF) will independently extract the following information after training:

(1) Basic information: Title, author, country, year, language, etc.
(2) Baseline information: Gender, age, number of persons, country, diagnostic criteria, etc.
(3) Methodological information: Grouping method, allocation concealment, blind method, result bias, etc.
(4) Intervention measures: Treatment measures, dosage, treatment time, frequency, etc.
(5) Results: Data of primary and secondary results.

After the work is completed, the results are cross-checked, if there are differences, a group discussion is conducted to determine the final result.

### 2.4 Assessment of risk of bias in included studies

The 2 authors (YT and YSF) evaluated the article methodology of inclusive trials independently, by the Cochrane collaboration “bias risk” tool sequences generated from six aspects of allocation concealment, blind (or mask), incomplete data evaluation, evaluation reports, and other sources of bias selective results. Finally, for each items, we will made ranking of “Low-risk bias,” “High-risk bias,” and “Unclear” based on the Cochrane collaboration “bias risk” tool.^[19,20]^

### 2.5 Data analysis

#### 2.5.1 Management of lost data

If data are insufficient from the selected study, we will contact the author via email for the required data. If baseline and outcome data or other data are included, the mean and standard deviation of the change will be manually calculated according to the Cochrane.^[21]^

#### 2.5.2 Network map

In the network diagram, each dot represents an intervention; The larger dot area means the bigger population of the studied intervention; The line between the 2 dots represents that there is direct comparison to RCT studies among 2 interventions; The line thickness represents the numbers of direct comparison to RCT studies among 2 interventions.

#### 2.5.3 Transitivity and Consistency Assessment

Transitivity and consistency are the prerequisites for reticular meta-analysis. The transitivity was evaluated qualitatively from the perspective of methodology and was evaluated according to the PICO principle. Consistency was mainly to check local and overall consistency. Local consistency can be checked by loop consistency test (Higgins model). The global consistency test was verified by the corresponding inconsistency model according to different data.

#### 2.5.4 Assessment of heterogeneity

Heterogeneity tests for all included studies were performed by using Network prediction interval graph, then to study the relationship of the weighted mean difference (WMD) at a 95% confidence interval (95% CI) and estimation zone (95%Prl) to invalid line, only when invalid line crosses perpendicularly to estimation zone but does not to CI, then it means that heterogeneity exists.^[22,23]^

#### 2.5.5 Pairwise meta-analysis

If there is a direct comparison between the experimental interventions included in the data (Injection therapy s Injection therapy, Injection therapy vs placebo), the Stata14.0 will be used for pairwise meta-analysis based on a random-effects model.

#### 2.5.6 Network meta-analysis

Two team members (YT and YSF) used statistical software Stata (version 14.0; Stata Corporation, College Station, TX) for analysis. A random effects model was used for network meta-analysis to compare the variables between different interventions. By comparing Surface Under the Cumulative Ranking Curve (SUCRA), the optimum intervention measures were determined. The range of SUCRA is 0% to 100%; the higher of the cumulative ranking curve means the better of the efficacy.

#### 2.5.7 Assessment of reporting biases

Funnel plots are used to detect publication bias. If the images are asymmetric, it indicates that there is publication bias.

#### 2.5.8 Subgroup analysis

If the analysis shows significant heterogeneity, then the root cause will be analyzed according to the PICOS principle, and the STATA 14.0 will be used for subgroup analysis.

#### 2.5.9 Grading the quality of evidence

According to the standards in the Grading of Recommendations Assessment Development and Evaluation (GRADE) system,^[24]^ 2 team members evaluate the quality of the research and divide it into 4 levels of “high,” “medium,” “low,” and “very low”; then the results will be exchanged. If there is any disagreement, the final option will be selected via group discussion.

### 2.6 Ethics and dissemination

The secondary literature study has no relationship to the personal data of the study, so the ethical approval is not required. Evaluation of the Comparative Efficacy and Safety of Injection therapies for Knee osteoarthritis may provide evidence for clinical treatment of this disease. The results of the study will be published in a peer-reviewed journal.

## 3 Discussion

Knee osteoarthritis is the main cause of lower extremity disability among senior people, and the main pathological changes are degenerative changes, destruction, and hyperosteogeny of articular cartilage.^[25,26]^ At present, the pathogenesis of this disease is unknown, but now, it is mostly considered that joint structure and function failure are caused by senile degeneration, osteoporosis, inflammation, metabolic factors, and so on.^[27]^ Compared with the surgical treatment, conservative method is more acceptable, and the clinical efficacy is also worthy of attention. However, IA therapy has become an optimized method for conservative treatment, which can reduce local pain and improve joint's function and activity by injecting drugs directly into the lumen of the knee.^[28]^ In face of multiple injections, it is our common concern to select a high effective drug without side effects. Therefore, the purpose of this study is to evaluate the efficacy and safety of multiple injection therapies in the treatment of knee osteoarthritis, so that to provide ideas, methods, and evidence for clinical intervention KOA.

In this study, first, the limitations were discussed according to the different injection therapies. Ignoring to the dose, injection position and time would also affect the efficacy.

Second, in terms of efficacy evaluation, we selected several criteria, among which VAS score was obtained according to the subjective feelings of patients, which was not objective.

Finally, many high-quality RCTs were excluded because of the stringent inclusion criteria, and the feasibility of the articles depends on the methodological quality of the included articles.^[14,15]^

## Author contributions

**Data curation:** ting yu, Shifan Yan.

**Formal analysis:** ting yu, Shifan Yan.

**Investigation:** Genping Zhong.

**Resources:** Daocheng Zhu.

**Software:** Daocheng Zhu, Siyu Qin.

**Supervision:** Zhenhai Chi, Xilin Ouyang.

**Validation:** Pan Cheng, Haiyan Li.

**Writing – original draft:** Rixin Chen.

**Writing – review & editing:** Lin Jiao.
